# Systemic sporotrichosis in an alcoholic patient^☆^^☆☆^

**DOI:** 10.1016/j.abd.2019.08.029

**Published:** 2020-03-20

**Authors:** Norami de Moura Barros, Allen de Souza Pessoa, Arles Martins Brotas

**Affiliations:** Department of Dermatology, Hospital Universitário Pedro Ernesto, Universidade do Estado do Rio de Janeiro, Rio de Janeiro, RJ, Brazil

**Keywords:** Alcoholism, Immunosuppression, Sporotrichosis

## Abstract

A 44-year-old male patient presented with nodules that evolved with inflammation, following drainage of seropurulent secretion and ulceration. The patient had a 6 year-history of alcohol addiction and reported contact with cats. At the physical examination, the patient had skin-colored and erythematous nodules, and ulcers covered with thick, blackened crusts on the face, trunk and limbs. A culture of a nodule fluid revealed growth of *Sporotrix* sp. He also had pulmonary involvement and therefore the disease was classified as systemic sporotrichosis, a rare form that usually affect patients infected with HIV. Chronic alcohol abuse was considered the factor of immunosuppression for the patient.

Sporotrichosis is a subacute or chronic subcutaneous mycosis caused by dimorphic fungi of the genus *Sporothrix*. Approximately 80% of the affected patients present the lymphocutaneous form. However, in patients with immunosuppression, disseminated forms can occur.^1^, ^2^

A 44-year-old male patient, presented with subcutaneous nodules that developed inflammation, following drainage of seropurulent secretion and ulceration. The patient had a 6 year-history of alcohol addiction. He denied comorbidities, although he reported prolonged contact with cats of unknown origin.

At the physical examination, the patient had skin-colored and erythematous subcutaneous nodules, and ulcers covered with thick, blackened crusts on the face, trunk and limbs (Figure 1, Figure 2).

**Figure 1:**
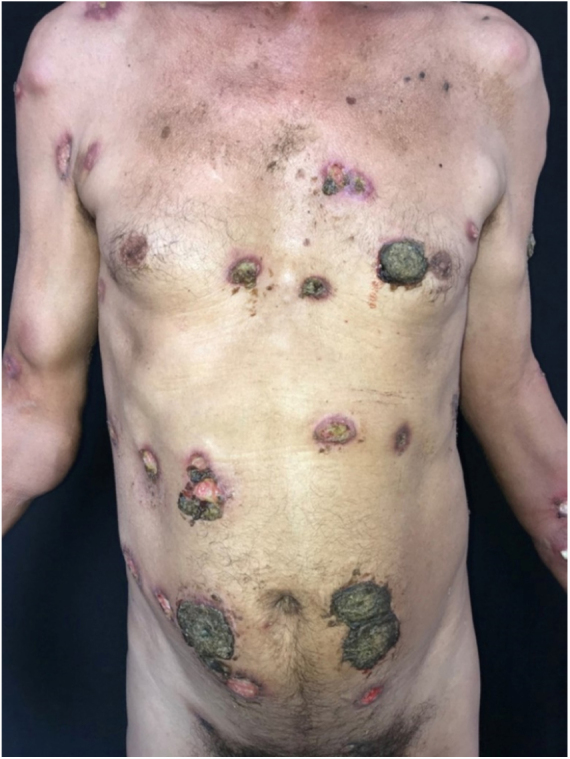
Skin-colored and erythematous nodules and ulcers covered with thick, blackened crusts.

**Figure 2:**
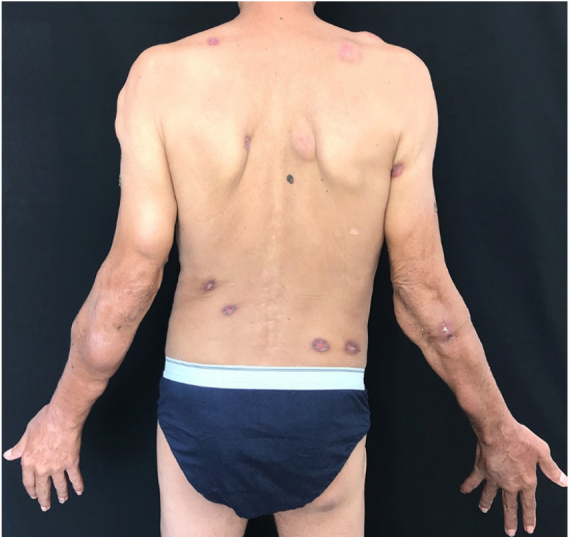
Skin-colored and erythematous nodules and ulcers covered with thick, blackened crusts.

Due to compatible epidemiological history and the evolution pattern of the lesions, the main diagnostic hypothesis was sporotrichosis.

A culture of the aspirated fluid of one of the nodules was performed, which revealed growth of *Sporotrix* sp. after 5 days (Figure 3, Figure 4).

**Figure 3:**
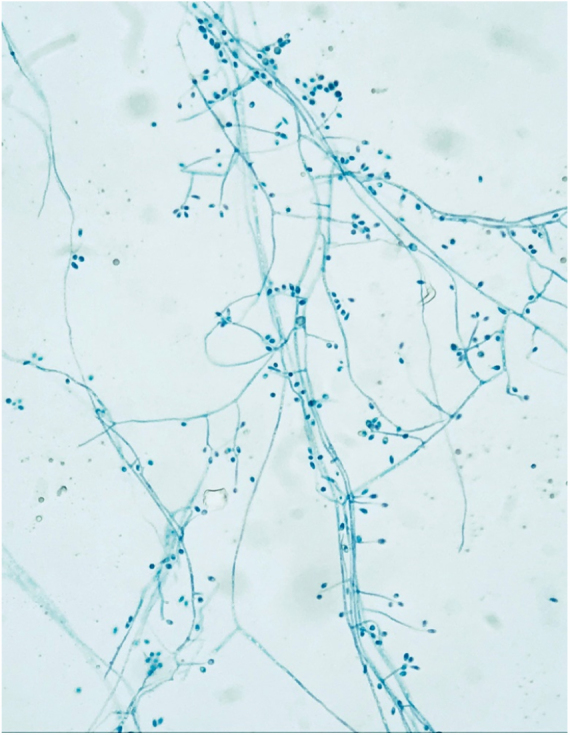
Hyaline, septate, branched and regular hyphae. Pyriform conidia arranged like a daisy flower at the end of the conidiophores.

**Figure 4:**
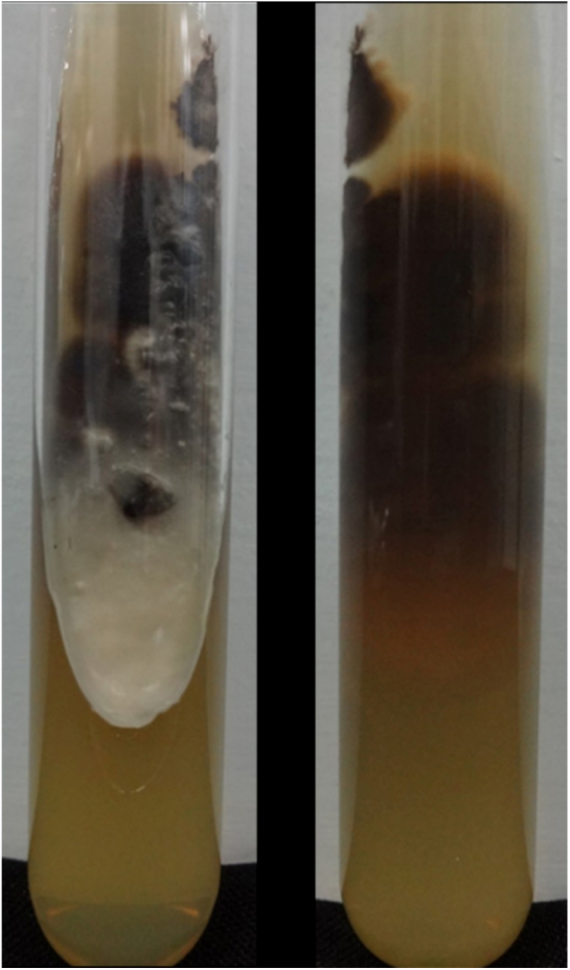
Membranous colony with white and blackened areas, and colorless back.

The laboratory tests (hemogram, kidney function, liver function and thyroid function) did not show any abnormal results, and the serology test results (hepatitis B, hepatitis C, VDRL, HTLV and HIV) were negative.

High-resolution chest CT revealed atelectasis, ground-glass infiltrate, hilar lymphadenopathy and pleural effusion in both lungs.

The disease was classified as systemic sporotrichosis, according to the classification recommended by Orofino-Costa et al,^3^ illustrating an exuberant presentation in a patient immunosuppressed by alcoholism.^3^

Patient was treated with amphotericin B lipid complex for 28 days, followed by itraconazole, during 11 months and had a good response, with healing of ulcers and without relapses in a 6-month follow-up.

The feline zoonotic transmission of sporotrichosis was observed in the 1990s in the State of Rio de Janeiro, Brazil, which is currently considered to be a hyperendemic area. In the South and Southeast Brazilian regions, *S. brasiliensis* is the main (88%) etiological agent of human and animal sporotrichosis.^3^

Systemic forms are rare and usually affect immunocompromised individuals, mostly those with HIV .^3^

Chronic alcohol abuse results in lymphopenia and chronic activation of the T-cell pool, which may alter the T-cell ability to expand and respond to pathogenic agents, inducing to an anergy state and, changing Th1 and Th2 response.^4^

Th1 response is considered as the main control factor of fungal infection. In addition to patient immunosuppression, we should emphasize that *S. brasiliensis* is the most virulent species of this genus, due to its ability to invade tissues and lead one to death.^3^, ^5^

The high prevalence of alcohol abuse in the Brazilian population, estimated at 13.7%, and the increasing zoonotic transmission of sporotrichosis may lead to an increase in the prevalence of disseminated forms of the disease.^6^

Our report corroborates the association previously reported by others between alcoholism and the spread of sporotrichosis.^7^, ^8^, ^9^, ^10^

## Financial support

None declared.

## Authors' contributions

Norami de Moura Barros: Approval of the final version of the manuscript; conception and planning of the study; elaboration and writing of the manuscript; obtaining, analysis, and interpretation of the data; intellectual participation in the propaedeutic and/or therapeutic conduct of the studied cases; critical review of the literature; critical review of the manuscript.

Allen de Souza Pessoa: Approval of the final version of the manuscript; conception and planning of the study; elaboration and writing of the manuscript; obtaining, analysis, and interpretation of the data; intellectual participation in the propaedeutic and/or therapeutic conduct of the studied cases; critical review of the literature; critical review of the manuscript.

Arles Martins Brotas: Conception and planning of the study; obtaining, analysis, and interpretation of the data; effective participation in research orientation; intellectual participation in the propaedeutic and/or therapeutic conduct of the studied cases; critical review of the literature; critical review of the literature.

## Conflicts of interest

None declared.
